# Combined spinal-epidural analgesia and epidural analgesia induced maternal fever with a similar timing during labor-A randomized controlled clinical trial

**DOI:** 10.3389/fmed.2022.927346

**Published:** 2022-08-09

**Authors:** Qinjun Chu, Yan Sun, Lihui Bai, Yafan Bai, Dongqing Zhang, Ping Zheng, Xiaogao Jin

**Affiliations:** ^1^Department of Anesthesiology and Perioperative Medicine, Zhengzhou Central Hospital Affiliated to Zhengzhou University, Zhengzhou, China; ^2^Department of Anesthesiology, The Maternal and Children Hospital of Zhengzhou, Zhengzhou, China; ^3^Delivery Room, The Maternal and Children Hospital of Zhengzhou, Zhengzhou, China; ^4^West Houston Family Practice, Houston, TX, United States; ^5^Metabolic Disease Research Center, Zhengzhou Central Hospital Affiliated to Zhengzhou University, Zhengzhou, China; ^6^Center for Advanced Medicine, College of Medicine, Zhengzhou University, Zhengzhou, China

**Keywords:** epidural [MeSH], fever, delivery, spinal, analgesia

## Abstract

**Background:**

Both epidural and combined spinal-epidural (EA and CSEA) analgesia can induce intrapartum maternal fever. CSEA has a more rapid onset and wider nerve block than EA. Therefore, CSEA might have a different profile of intrapartum maternal fever, including higher temperatures or earlier occurrence. This randomized clinical trial was to determine whether CSEA could cause maternal fever earlier than EA.

**Methods:**

A randomized, double-blind, controlled clinical trial was performed on 233 nulliparous full-term pregnant women during vaginal delivery. The pregnant women were randomly allocated into the EA group (*n* = 113) and the CSEA group (*n* = 120). The fever latent period, from analgesia start to fever occurrence, was the primary endpoint in this study. The temperature was measured every 30 min using an eardrum thermometer during labor analgesia. The fever was defined as an eardrum temperature of ≥38 °C.

**Results:**

No difference was found in the maternal fever rate between the EA and the CSEA groups (10/113 vs. 7/120, *P* = 0.356). There was no significant difference in the fever latent period between the two groups (4.75 ± 0.86 h vs. 3.79 ± 2.2 h, *p* = 0.305). The temperatures at all points had no differences between EA and CSEA.

**Conclusion:**

CSEA had a similar latent fever period as EA. A further study is warranted to confirm the similar characteristic between CSEA and EA in the development of intrapartum maternal fever.

**Clinical trial registration:**

www.chictr.org.cn, identifier ChiCTR2000038793.

## Introduction

Both epidural and combined spinal-epidural analgesia (EA and CSEA) are widely used to provide pain relief during labor (1–6). However, approximately 6.6–46.3% of pregnant women with epidural labor analgesia will experience intrapartum fever, which is called epidural-related maternal fever (ERMF) (7). ERMF was proved to be caused by a non-infectious maternal inflammation which was manifested by pro-inflammatory cytokines increase in mother serum (8–10). It was still unclear how the inflammation is caused by EA during vaginal delivery since ERMF was first described by Fusi et al. (11), White et al. (12), and Sharpe and Arendt (13). ERMF is paid more and more attention recently because ERMF is a kind of maternal immune activation that has been reported to contribute to neuronal damage in fetal brain and be related to schizophrenia, seizure, and autism (1, 8, 14–16). Maternal intrapartum fever may come from the infections, such as chorioamnionitis, or a non-infectious factor, such as ERMF. The pregnant women with intrapartum fever have a chance of up to 50% of receiving antibiotic therapy and a higher risk of operative delivery (17). The neonates from the febrile mothers were more usually admitted to the Neonatal Intensive Care Unit where they will be given prophylactic antibiotics for the suspected sepsis (18). The prolonged duration of labor is a risk factor for ERMF) (19). Therefore, the maternal intrapartum fever will not be considered as ERMF if it occurs during the early labor after analgesia. CSEA can more rapidly relieve the pain than EA. So, it is possible that the febrile patients with CSEA may have a greater chance of being diagnosed with infection and receiving unnecessary antibiotic therapy than the patients with EA. So, it is very important to identify the difference in the timing of fever between CSEA and EA to differentiate the diagnosis of ERMF.

Evidence indicated that CSEA induces a similar ERMF as EA (20). The rapidity of nerve block may or may not influence the timing of the onset of ERMF. Further, stimulation of both the spinal and epidural spaces simultaneously may trigger a more robust inflammatory response if this is the mechanism for ERMF. In either case, we hypothesize that the onset of maternal temperature increase may be more rapid with CSEA compared to EA. Earlier maternal fever may be more possible to diagnose as an infection and trigger an unnecessary Cesarean section. Therefore, it is important to confirm whether CSEA really induced an earlier maternal fever than EA. So far, there was no direct evidence about the difference in effects on intrapartum maternal fever between epidural and CSEA. Here, we hypothesize that CSEA can induce an earlier maternal fever than EA. The objective of the present study was to determine whether CSEA induces intrapartum maternal fever earlier than EA.

## Methods

### Research population

This prospective, randomized, clinical trial study was conducted at Zhengzhou Central Hospital and The Maternal and Children's Hospital of Zhengzhou from March to May 2021. This study was approved by the medical ethics committee (Zhengzhou Central Hospital Affiliated to Zhengzhou University, Zhengzhou, China) with approval number 202118. The study protocol was registered in the Chinese Clinical Trial Registry with the study registration number: ChiCTR2000038793. Inclusion criteria were being aged 18–40 years, ASA physical status I or II, nulliparous women, at term, singleton, with regular contractions, and cervical dilation of 3 cm or more. Patients with spinal disease, the presence of underlying diseases such as hypothyroidism or hyperthyroidism, high-risk pregnancies (placenta previa, placental abruption, severe preeclampsia/eclampsia, and HIV-positive patients), were excluded from the study. All included patients agreed to participate in the clinical trial and signed an informed consent form. The eligible pregnant women were randomized into two groups, namely, the epidural group and the CSEA group. The block randomization with a block size of 4 was performed using the R-software-generated random list prepared by an independent research nurse who was not involved in the rest of the investigation. Based on the list, sealed, opaque, sequentially numbered envelopes containing cards with the allocation information were prepared. Upon the eligible parturients' arrival to the labor and delivery room, the investigator nurse assigned to the participants by opening the envelopes containing the pre-written assignment.

### Sample size

The primary endpoint of this study was the latent fever period, defined as the time from the beginning of analgesia to fever (≥38°C). The maternal fever was assumed to be observed within 2 h after CSEA started; however, the fever in EA was expected to occur in the fifth hour after analgesia according to the reported literatures. So, the accepted effect size was determined as 3-h difference between the CSEA and epidural groups in this study. The sample size was calculated as 7 patients in each group using the parameters alpha = 0.05, *Z*_alpha_ = 1.96, power = 80%, *Z*_beta_ = 0.84, expected SD = 2 h, accepted effect size = 3 h (21). Considering an incidence of maternal fever of 6.6–30% for ERMF (5), the fever incidence of 10% was used to calculate the sample size for each group. A sample size of 70 patients would be required in each group according to calculation. In order to compensate for any exclusion that might occur after randomization, this sample size was estimated to be about 80 patients in each group. In real practice, enrollment would be closed until each group successfully achieved 7 parturients with fever during delivery.

### Blind design

Anesthesiologists were unblinded to the assignment and administered epidural or CSEA blocks according to the research protocol. The nurses who took the temperatures were unaware of group assignments during the research. Research assistants responsible for data collection and analysis were blinded to group assignments. The parturients were also blinded as epidural or CSEA block was performed. Study enrollment ceased when the target sample size was obtained.

### Preparation before analgesia

The attending obstetrician determined those who could deliver vaginally and enter the labor and delivery room by evaluating the regular contractions of each pregnant woman. An intravenous (IV) line was established with an 18-gauge IV cannula on the forearm. Ringer's lactate (RL) solution at a rate of 2–4 ml/kg/h was used to maintain the basal fluid. Rescue vasoactive agents including atropine, ephedrine, or epinephrine were available at the bedside. Maternal vital signs (non-invasive BP, heart rate, and respiratory rate) were monitored and fetal heart rate (FHR) monitoring is also used routinely in labor. Neuraxial labor analgesia (epidural or CSEA) was performed when contractions became regular and cervical dilation was ≥ 3 cm.

### Epidural and CSEA protocol

The pregnant women were placed in a lateral decubitus position. The epidural puncture was performed using an 18 G Tuohy needle at the L3-4 intervertebral space, and then an epidural catheter was introduced through the needle. A test dose of 10 mg/ml 3 ml containing 0.005 mg/ml epinephrine was administrated by the epidural catheter to rule out the possible placement in subarachnoid space or vessel. After 5-min observation, an initial dose of 10 ml solution containing 10 mg ropivacaine and 5 μg sufentanil was administrated through an epidural catheter. Then, the epidural catheter was connected to an electronic patient-controlled analgesia (PCA) pump with the following parameters: a continuous background infusion of the mixture containing ropivacaine 0.08% plus sufentanil 0.4 μg/ml at 8 ml/h; loading dose, 8 ml containing the same analgesic mixture; lockout interval, 15 min; maximum dose, 32 ml/h. CSEA was performed by a needle-through-needle technique after an epidural puncture. Spinal administration was 3 mg of ropivacaine and 1.5 μg of sufentanil in 3 ml of saline. The epidural component in the CSEA group was the same as the epidural protocol.

### Pain evaluation and management

A visual analog scale (VAS: 0 = no pain; 10 = the worst pain imaginable) was used to evaluate pain severity during labor. If the analgesia protocol above was inefficient to control labor pain, a rescue dose of 5 ml solution containing 0.8 mg/ml ropivacaine and 0.4 μg/ml sufentanil was injected to improve labor pain via the epidural catheter.

### Body temperature measurement

The maternal temperature was measured and recorded by the delivery room nurse every half an hour throughout the labor using a tympanic probe (ThermoScan3, Infrared ear thermometer, Braun, Germany). The delivery room temperature was maintained between 24 and 26 °C. To ensure accurate temperature measurement, all the nurses taking the temperature received standard training about the use of the thermometer. The nurse taking the temperature was unaware of group assignments during research. The fever was defined as an eardrum temperature of ≥38°C. Moreover, to compare the fever latent period related to labor analgesia in two groups, we defined the fever latent period as the time from medication administration to fever occurrence in the EA group, and from intrathecal injection of local anesthetics to fever occurrence in the CSEA group. If the parturient developed a fever, the following measures were taken: when the tympanic temperature was between 38°C and 38.5°C, pregnant women were encouraged to drink more water, and physical cooling was used. Stages of labor were judged by the obstetrician. The first phase of labor began with the onset of regular contractions and ended when the cervix was fully dilated. The second phase of labor began when the cervix was fully dilated and ended with the delivery of the neonate. The third phase of labor began with the delivery of the neonate and ended with the delivery of the placenta.

### Statistical analysis

Continuous variables were compared using the independent-samples *T*-test for normally distributed data or Mann–Whitney *U*-test for non-parametric data. Categorical variables were compared using Chi-Square or Fisher's exact test as appropriate. Change in temperature over time was evaluated with repeated measures analysis of variance, followed by Bonferroni correction with appropriate adjustments in *P* values for multiple comparisons. Statistical significance was defined for an overall error at the 5% level. All analyses were performed using R software (22).

## Results

### Participants enrolled in this study

**Figure 1:**
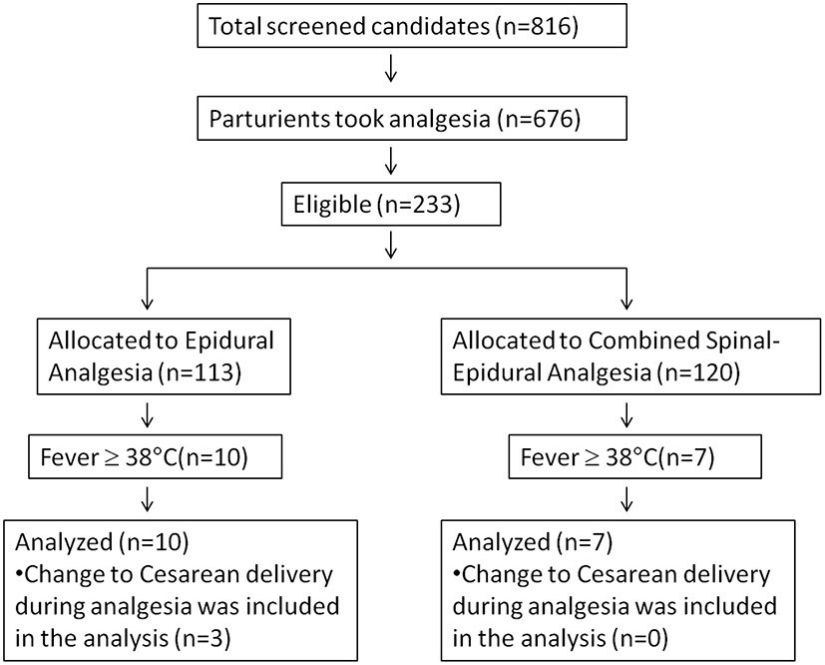
Flow chart of the participants in the study.

We had overestimated the incidence of epidural-related maternal fever as 10% during power analysis. The real incidence of maternal fever was 7.3% in this study. Therefore, we had to enroll more patients to get at least 7 cases of fever in each group. Finally, 233 pregnant women were enrolled in this study, which were randomized into the EA group (*n* = 113) and the combined spinal-EA group (*n* = 120) (Figure 1). Ten participants in the EA group experienced fever during labor, and seven participants in the combined spinal-EA group experienced fever. To compare the fever rate of the two groups, the Chi-square test was used without any exclusion and no difference was found (10/113 vs. 7/120, *P* = 0.376). There were three participants with fever in the epidural group who were transferred to cesarean delivery because of a diagnosis of chorioamnionitis and included in the final analysis.

### The characteristics of all participants in the two groups

The characteristics of all participants at admission in both groups were compared to assess the homogeneity. There were no significant differences in age, weight, height, gestational age, or cervical dilation in both group before analgesia (Table 1). The EA group had a longer duration of 1st stage than the CSEA group (*p* < 0.036). The combined spinal-epidural group, with less sufentanil dose, ropivacaine, total volume, and PCA press times, showed more analgesia effects than the epidural group.

**Table 1:** Characteristics of all the parturients at admission and the delivery outcomes after labor.

**Variable**	**Epidural (*n* = 113)**	**Combined^δ^ (*n* = 120)**	***P* value**
Age (years)	30 ± 3	31 ± 3	0.06
Weight (kg)	67 ± 3	68 ± 3	0.97
Height (cm)	160 ± 3	162 ± 3	0.85
Gestation (Week)	39.62 ± 0.87	39.71 ± 0.71	0.77
Cervical dilation (cm)	3.2 ± 0.8	3.4 ± 1.1	0.88
White blood cell count (10^9^/L)	8.59 ± 2.00	8.76 ± 2.29	0.75
Fever (Number)	10	7	0.38^Δ^
Cesarean delivery (Number)	8	7	0.69^Δ^
Duration 1st stage (min)	280 ± 179	233 ± 120	0.036
Duration 2nd stage (min)	46 ± 26	51 ± 43	0.386
Duration of membrane rupture (min)	92 ± 136	75 ± 183	0.754
Vaginal examination times*	5(5,7)	6(5,8)	0.086^#^
Oxytocin administration (Number)	65	69	1^Δ^
Instrumental delivery (Number)	0	0	-
Birth Weight (g)	3,352 ± 370	3,352 ± 370	0.713
Apgar score at 1 min	9	9	-
Apgar score at 5 min	10	10	-
Sufentanil dose (μg)	31 ± 10	29 ± 8	0.034
Ropivacaine (mg)	62 ± 19	57 ± 16	0.034
Total volume (ml)	65 ± 24	55 ± 20	0.001
PCA press times*	2(1, 3)	1(1, 2)	0.012^#^
Hypotension (Number)	0	0	-
Pruritus (Number)	0	0	-

^*^Median (25th,75th);

^#^Analyzed by Kruskal–Wallis H test;

^Δ^Chi-square test.

^δ^Combined means combined spinal-EA.

### Maternal and neonatal outcomes in the two groups

For the parturients with fever in the two groups, no differences were found in the outcomes of the pregnant women and neonates, including side effects. However, the parturients with fever in the EA group had an older age than the CSEA labor analgesia (Table 2).

**Table 2:** Characteristics of the parturients with fever at admission and the delivery outcomes after vaginal delivery.

**Variable**	**Epidural (*n* = 10)**	**Combined (*n* = 7)**	***P* value**
Age (years)	30.4 ± 2.4	24.9 ± 3.2	0.002
Weight (kg)	68.07 ± 8.03	70.71 ± 10.38	0.604
Height (cm)	162.86 ± 4.67	164.00 ± 2.7	0.586
Gestation (Week)	39.45 ± 0.98	39.82 ± 0.81	0.450
Cervical dilation (cm)	3	3	-
White blood cell count (10^9^/L)	8.00 ± 1.30	9.59 ± 3.67	0.314
Duration 1st stage (min)	367 ± 229	382 ± 207	0.902
Duration 2nd stage (min)	57 ± 39	46 ± 50	0.318
Vaginal examination times	7.00 ± 2.16	6.43 ± 2.23	0.635
Oxytocin administration (Number)	4	4	-
Instrumental delivery (Number)	0	0	-
Birth Weight (g)	3,153.71 ± 199.74	3,330.86 ± 359.77	0.28
Apgar score at 1 min	9	9	-
Apgar score at 5 min	10	10	-
Sufentanil dose (μg)	38 ± 5	34 ± 6	0.212
Ropivacaine (mg)	75 ± 10	68 ± 11	0.212
Total volume (ml)	82 ± 13	69 ± 14	0.092
PCA press times	2.9 ± 1.2	2.0 ± 0.8	0.147
Hypotension (Number)	0	0	-
Pruritus (Number)	0	0	-

### Temperature changes over time during labor analgesia

**Figure 2:**
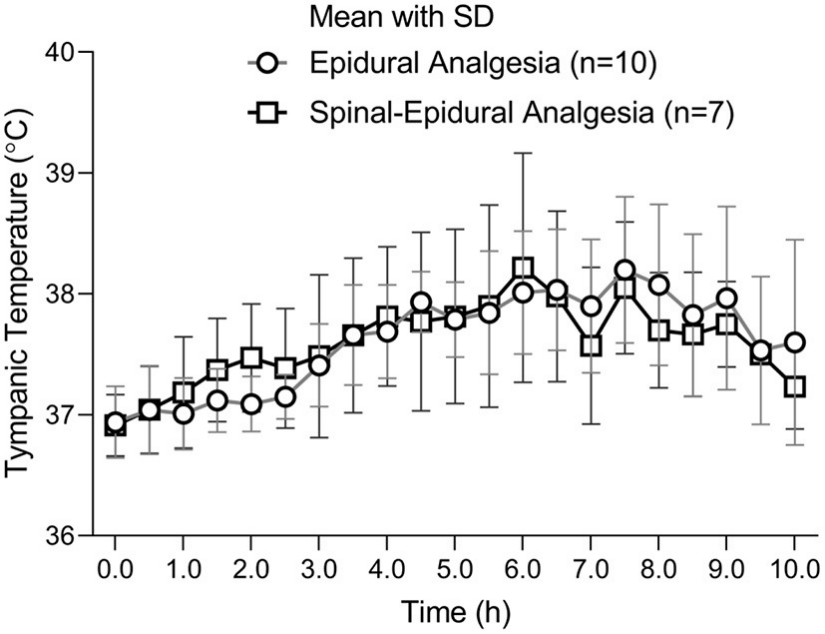
The tympanic temperature changes over time.

A curve of maternal mean temperature over time was established to demonstrate the fever development in the pregnant women during labor analgesia (Figure 2). There was no significant difference at all time points between the two groups (Figure 2).

The changes of mean temperatures with the standard error were displayed on the every 0.5 h time points. There was no difference between the two groups at all time points. The number of parturients was 10 in the EA group and 7 in CSEA group.

### Temperature changes over different events points

**Figure 3:**
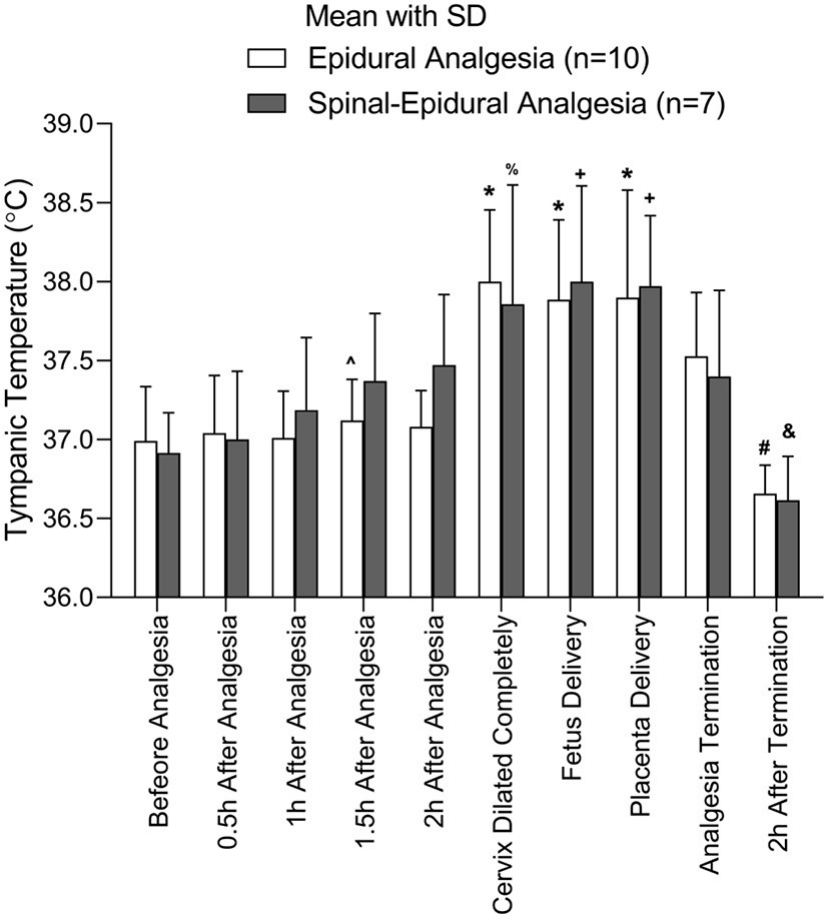
The temperature changes over different events points.

Because each pregnant woman has a different vaginal delivery course, the participants may have different major events at a certain time point. To properly compare the temperature at different major events, the temperature at the major events was analyzed between the two groups. The major events in the analysis included before analgesia, 0.5 h after analgesia, 1 h after analgesia, 1.5 h after analgesia, 2 h after analgesia, full cervical dilatation, fetus delivery, placenta delivery, analgesia termination, and 2 h after termination. No differences were found in the temperatures at the major events between the two groups. The temperature for the participants with fever would reach a ceiling when the cervix dilated completely and form a plateau from the complete cervix dilation to placenta delivery (Figure 3).

The comparison of tympanic temperature between epidural and combined spinal-EA at the different event points. ^*^Compared to before analgesia, 0.5 h after analgesia, and 1 h after analgesia, *p* < 0.05; ^#^Compared to cervix dilated completely, fetus delivery, placenta delivery, and analgesia termination, *p* < 0.05. ∧Compared to cervix dilated completely, *p* < 0.05. ^+^Compared to before analgesia, 0.5 h after analgesia, *p* < 0.05; ^%^Compared to before analgesia, *p* < 0.05; ^&^Compared to 2 h after analgesia, cervix dilated completely, fetus delivery, and placenta delivery, *p* < 0.05. The number of parturients was 10 in the EA group and 7 in the CSEA group.

### The fever latent period in the two groups

**Figure 4:**
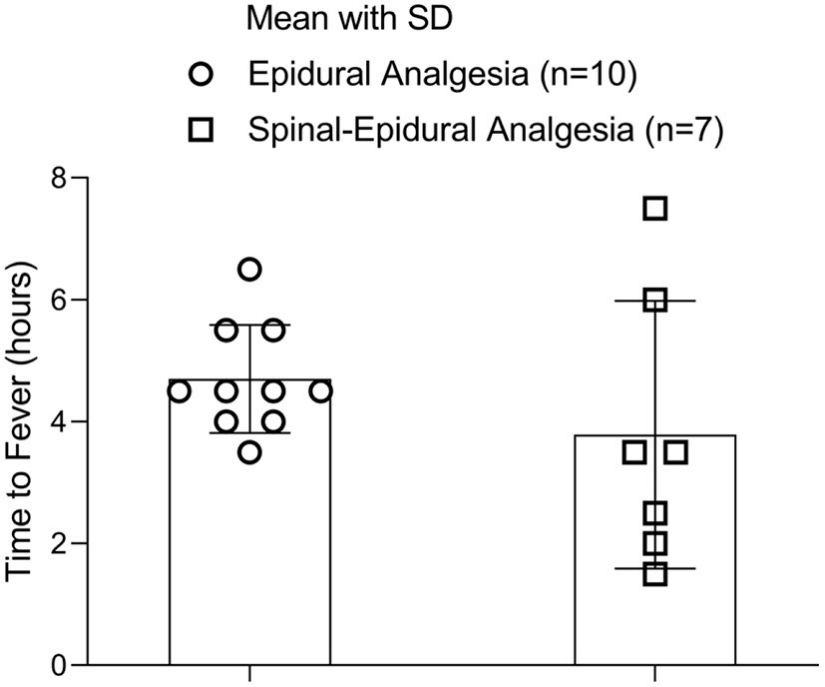
The fever latent period.

There was no significant difference in latent fever period-related labor analgesia between the two groups (4.75 ± 0.86 h vs. 3.79 ± 2.2 h, *p* = 0.305) (Figure 4).

The number of parturients was 10 in the EA group and 7 in the CSEA group.

## Discussion

In the present study, we found that CSEA had a similar fever latent period as EA. There was also no difference in temperatures between the two methods at any time points.

This study presented a negative result regarding the effects of CSEA and EA on fever latent period. In this study, CSEA and EA had no influence on both intrapartum maternal fever incidence and the latent fever period. It suggested that CSEA and EA shared the same mechanism in the development of ERMF even though CSEA was performed by an additional spinal medication. The latent fever period of CSEA varied wider (from 1.5 to 7 h after analgesia) than that of EA (from 3.5 to 6 h after analgesia) (Figure 4). This difference may result from the faster action and wider nerve block of CSEA. However, the range differences had no effect on the development of intrapartum maternal fever in this study. Some indirect evidence showed that the intrapartum maternal fever was higher in temperature in CSEA than in the EA. But this literature did not compare CSEA with EA in the same study. This study was the first clinical trial to investigate the difference in intrapartum maternal fever between CSEA and EA in the same study.

As mentioned above, CSEA and EA may have the same mechanism in the development of ERMF according to our negative results. Heat dissipation decrease was thought to be one of the mechanisms of ERMF. If it is the case, CSEA should demonstrate higher maternal fever incidence or a shorter latent fever period than EA. However, the results showed no difference between CSEA and EA. The negative results suggested that heat dissipation decrease was not the main mechanism of ERMF. Lots of evidence has proved that ERMF results from a high serum concentration of pro-inflammatory cytokines such as IL-6, IL-1_beta_, and TNF_alpha_ (9). It has been proved that the increases in cytokines were induced by non-infectious factors (10, 23–25). Local anesthetics were thought to be able to induce the production of these pro-inflammatory cytokines by the injuries they caused to human umbilical vein endothelial cells and human placental trophoblasts (26). Therefore, heat dissipation may be a limiting factor in the development of intrapartum maternal fever.

There were some pitfalls to the negative results of this study. First, the sample size may be too small to detect the difference in the latent fever period. This study was designed to examine the difference of 3 h in the latent fever period between the two groups. So, the sample size of 7 in each group would not be enough to detect the difference in this study if the real difference is <3 h. Second, this study detected the fever latent period but ignored how high the maternal temperature was when the fever was observed. We might miss the fact that CSEA may cause a higher maternal temperature than EA during intrapartum maternal fever. Third, the ages of the CSEA group were younger than those of the epidural group even after a randomized design. This age difference may cover the difference in the latent fever period between EA and CSEA. Fourth, we try to set up blindness for the research assistant who was responsible for data collection. However, it is not a real blindness because the methods of analgesia could be identified by the onset of pain relief. It is difficult to eliminate the subject bias from the observers, which may influence the conclusion of this study.

## Conclusion

CSEA had a similar latent fever period as EA. Moreover, the intrapartum maternal fever incidence and the temperatures at the time points had no differences between epidural and CSEA. A further study may be warranted to investigate the difference between CSEA and EA in maternal fever development.

## Data availability statement

The original contributions presented in the study are included in the article/supplementary material, further inquiries can be directed to the corresponding authors.

## Ethics statement

The studies involving human participants were reviewed and this study was approved by Medical Ethics Committee (Zhengzhou Central Hospital Affiliated to Zhengzhou University, Zhengzhou, China) with the approval number of 202118. The patients/participants provided their written informed consent to participate in this study.

## Author contributions

QC: conceptualization, investigation, resources, supervision, project administration, and funding acquisition. YS, LB, and YB: methodology. XJ: software, validation, data curation, and writing—review and editing. XJ and QC: formal analysis. PZ: writing—original draft preparation. YS, LB, and DZ: visualization. All authors reviewed and approved the manuscript.

## Conflict of interest

The authors declare that the research was conducted in the absence of any commercial or financial relationships that could be construed as a potential conflict of interest.

## Publisher's note

All claims expressed in this article are solely those of the authors and do not necessarily represent those of their affiliated organizations, or those of the publisher, the editors and the reviewers. Any product that may be evaluated in this article, or claim that may be made by its manufacturer, is not guaranteed or endorsed by the publisher.
